# Screening and identification of Lactic acid bacteria from Ya’an pickle water to effectively remove Pb^2+^

**DOI:** 10.1186/s13568-018-0724-y

**Published:** 2019-01-19

**Authors:** Derong Lin, Hongfu Cao, Yixin Zhong, Yichen Huang, Jinpeng Zou, Qi He, Ran Ji, Tao Qin, Yuan Chen, Dan Wang, Zhijun Wu, Wen Qin, Dingtao Wu, Hong Chen, Qing Zhang

**Affiliations:** 1 College of Food Science, Sichuan Agricultural University, Ya’an, 625014 Sichuan People’s Republic of China; 2 College of Mechanical and Electrical Engineering, Sichuan Agricultural University, Ya’an, 625014 China

**Keywords:** Pickle water, Lactic acid bacteria, Pb^2+^, Adsorption

## Abstract

Heavy metal lead, which enters the human body through food intake, endangers human health. Microbe has the ability of adsorbing heavy metal, among which lactic acid bacteria are promising microbes to adsorb and remove Pb^2+^. The purpose of this study was to screen lactic acid bacteria from Ya’an pickle water to effectively remove Pb^2+^. The 7 strains having strong ability to effectively remove Pb^2+^ were detected. These strains were identified by microscopic examination and 16S rDNA sequencing, 4 strains of *Lactobacillus plantarum* and 3 strains of *Lactobacillus brevis* were obtained. Then the bacteria had a blind adsorption effect on Pb^2+^. After microwave digestion, the Pb^2+^ concentration was measured by flame atomic absorption spectrometry. The highest removal reached 82.25%. The adsorption mechanism of lactic acid bacteria was mainly divided into biosorption and bioaccumulation. The 7 strains of lactic acid bacteria could provide potential for detoxification of contaminated foods and reduction of the Pb^2+^ accumulation in the human diet and animal feed. At the same time, this study was helpful to further understand the mechanism of Pb^2+^ being adsorbed by lactic acid bacteria.

**Electronic supplementary material:**

The online version of this article (10.1186/s13568-018-0724-y) contains supplementary material, which is available to authorized users.

## Introduction

Currently, pesticide residue, veterinary drugs residue and heavy metal pollution are blamed for the food safety incident. The pesticide and veterinary drugs residues in food may be reduced by pretreatments, such as heat blanching, brine rinsing and peeling. But heavy metal pollution is difficult to handle. Once the food is contaminated by heavy metal, it means it can only be destroyed because of the nonbiodegradability and accumulation of heavy metals (Ekere et al. 2016). Lead, as an important industrial raw material, has been widely applied to modern industry including metallurgy, printing, military, medicine, electronics, ceramics, pigment and transportation industry (Özcan et al. 2009), but it is also toxic heavy metal. The lead ion (Pb^2+^) is one of the sources of environmental pollution. It has various sources and high cumulative toxicity, which can cause serious harm to human nervous system, hematopoietic function of bone marrow and so on, and environmental problems of ecosystems such as air pollution, brine pollution and soil pollution (Cui et al. 2017). Consequently, removing Pb^2+^ has aroused more people’s attention.

The traditional approaches for removing Pb^2+^ are precipitation, coagulation, adsorption and ion exchange. The ion exchange was applied for the selective removal of Pb^2+^ from waste brine samples. The polyacrylamide zirconium (IV) hybrid cation exchanger has a high selectivity to Pb^2+^ in comparison to other metal ions (Rahman et al. 2013). With the development of the technology, the use of ceramsite (Wang et al. 2018), ferric oxide (Saha et al. 2014) and other substances to adsorb heavy metals has also appeared. Ferric oxide has been widely used in the treatment of Pb^2+^ in waste brine (Zhang and Li 2016), but it was not suitable to use the iron oxide to fill in the pipe column directly, which was poor permeability and low efficiency. In general, most of these approaches required special equipment and rigorous experimental condition, which greatly limited their practically applicable value (Kobya et al. 2005). Recently, biosorption, a new method to eliminate heavy metal pollution, has been researched as a popular topic because of the advantages of low investment, high efficiency and safety with no side effects (Tural et al. 2017). Therefore, biosorption was important to remove Pb^2+^ from the aqueous solution selectively and efficiently.

Many reports commonly showed the biosorption of heavy metals by bacteria and fungi. Iskandar et al. reported the biosorption by filamentous fungi isolated from a fresh brine ecosystem (Iskandar et al. 2012), and there were some reports showed that pretreating *Aspergillus niger* would significantly improve the biosorption of Pb^2+^ (Kapoor and Viraraghavan 1998). While, some bacteria used for biosorption may be pathogenicity, which was easy to cause secondary pollution. More reports showed that lactic acid bacteria (LAB) strains were probiotic bacteria which were acknowledged as the safety level microorganisms which were widely used to produce fermented food (Teusink and Molenaar 2017; Jahromi et al. 2017), and were also applied into heavy metal biosorption. Schut et al. found that copper could be removed by biosorption of wine-relevant lactobacilli (Schut et al. 2011). Besides, some LAB strains were evaluated and selected through biosorption of cadmium, arsenic and mercury by Kinoshita et al. (Kinoshita et al. 2013). However, researches on the adsorption of LAB are still relatively rare.

In our previous studies, we have reviewed that the progress in mechanism and influence of biosorption between LAB and Pb^2+^ (Lin et al. 2017). Due to the probiotic role of the LAB, the security of adsorption with LAB would be higher than other methods of removing Pb^2+^. Considering the fact that LAB were extensive in pickle brine (Zafar et al. 2018), high-quality pickle brine was selected from Ya’an, Sichuan  (Xia et al. 2017), and expected to screen and identify LAB with strong adsorption capacity for Pb^2+^, and provided a microbiological method for further solving contamination in food.

## Materials and methods

### Materials and reagents

Pb^2+^ solutions were prepared from PbNO_3_ (Hangfeng Chemical Co., Inc., Chengdu, China) for using in different experiments. 0.85% physiological saline, 6% nitric acid (HNO_3_), hydrogen peroxide (H_2_O_2_), 25% potassium iodide solution (KI), ascorbic acid solution (AA), and methyl isobutyl ketone (MIBK) were purchased from Sigma–Aldrich (Shanghai, China). All solutions were prepared using analytical-grade reagents and water was distilled and deionized with a Milli-Q^®^ system (Millipore, Billerica, MA, USA).

### Sampling

Three samples of locally ripened Ya’an pickle brine were collected in July of 2015 from three different local farmers in Ya’an, Sichuan, China (every farmer provided 1 sample with 10 mL brine). The containers used for storing pickle brine were shaken prior to sampling. The samples were collected at ambient temperatures and kept on ice during the 3 h transport to our laboratory. The pickle brine from each sample was aseptically collected for further analysis.

### Isolation and screening of resistant bacteria

3 samples were exerted appropriate tenfold serial dilutions (10^−1^ to 10^−4^) with sterile PS, respectively. Every dilution of 50 μL was inoculated in MRS agar plate by spread plate method. After the incubation for 24 h at 37 °C, by the flat colony counting method, the plates colony number of 30–300 were selected out as the mother plates (TMP). Colonies were randomly selected from TMP. After purification and isolation, some suspected LAB colonies were inoculated respectively on the selective MRS plates (TSP) containing 2 mL 200 g/L Pb^2+^ by the plate streak. After then, TSP were incubated for 24 h at 37 °C. Some colonies of the TSP were selected out for acclimatization of bacteria.

They were inoculated in the De Man, Rogosa, and Sharpe (MRS) agar plate (specific for LAB) with 2 mL solution containing Pb^2+^ respectively (500 mg/L, 1000 mg/L, 1500 mg/L, 2000 mg/L) (Pb^2+^ medium) and to be isolated the strains which were capable of tolerating high concentration of Pb^2+^. After culture for 48 h at 37 °C,the dominant Pb^2+^-resistant strains were isolated from Pb^2+^ medium, and preserved with 30% glycerin at − 20 °C. These strains would be marked for subsequent identification.

### Identification of Pb^2+^ resistant bacteria

The morphological characteristics of the isolated strains of LAB were observed and recorded before and after Gram staining (Michael 1983).

The genotypic characterization of strains was performed by sequencing the gene 16S rDNA (Sontakke et al. 2009). Omega’s DNA extraction kit was used for total genomic DNA extraction, when strains grew to their late log phase. Fragments of bacterial 16S rDNA were amplified by polymerase chain reaction (PCR) using the primers 27F (5′-GCCTGTGCGGGGTGCTATAC-3′) and 1492R (5′-CGCCGTTGGCGGCGTGCTA-3′) (Bioengineering Co., Inc., Shanghai, China) with the thermocycler, whose accession number is MH681598, MH681599, MH681600, MH681601, MH681602, MH681603 and MH681604 respectively. *Lactobacillus plantarum* (*L. plantarum*) (MH681599) GDMCC 11516 is a *L. plantarum* strain that has been deposited in the culture collection ‘Guangdong Microbial Culture Center’, acronym GDMCC, the strain’s registration number is GDMCC 11516. PCR amplification products were sequenced by Sanger chain termination method and spliced by Contig Express splicing program.

The reaction parameters of PCR included five min of denaturation at 95 °C, followed by 35 cycles of 95 °C, for 30 s, 58 °C, for 30 s, 72 °C, for 90 s, and a final extension at 72 °C, for 7 min. After the reaction, the PCR products were subjected to 1% agarose gel electrophoresis to confirm the PCR amplification fragments. The PCR products were recovered by using the Omega’s DNA extraction kit (Omega Bio-tek, Co., Inc. USA). The purified PCR products were sent to Shanghai Bioengineering Limited by Share Ltd to sequence. Finally, the spliced sequence file was compared with the data in the NCBI ribosomal DNA sequence (Bacteria and Archaea) database by using the NCBI Blast program, and the strain information with the largest similarity to the sequence of the tested bacteria was obtained (Emel et al. 2017). The phylogenetic tree was analyzed and constructed with the neighbor-joining method (Gitzendanner et al. 2018).

### Bacterial adsorption assay

Those resistant strains were activated 2 times in 5 mL MRS broth at 37 °C for 24 h. Then amplification culture was in 10 mL MRS broth at 37 °C for 24 h. The cultured stains were centrifuged (4000 rpm, 4 °C, 15 min) and then washed with sterile PS for 2 times to get the wet bacteria.

Adsorption experiments were carried out on the basis of 3 g/L (the weight of wet bacteria/the volume of 200 mg/L Pb^2+^ solution). All the samples were exerted shake cultivation for 1 h at 37 °C, pH close to neutral. After incubation, the suspension of samples was centrifuged (4000 rpm, 4 °C, 15 min). A sample was taken from the supernatant to be digested.

All glass instruments and polytetrafluoroethylene tank (digestion tank) were soaked with 6% HNO_3_ for 24 h at room temperature, and then washed by ultrapure water for 2–3 times, then dried. The solvent was added to every sample and two reagent blanks, meanwhile every sample need a parallel experiment. All the digestion experiments were set at 600 watts (W). Microwave digestion instrument (MDI, CEM Co., Inc., USA) was started in strict accordance with the operating instructions (Acar et al. 2016).

According to microwave digestion program (Additional file 1: Table S1) and the setting of solvent proportion (Additional file 1: Table S2), the sample was digested and the optimum proportion of solvent was determined. After the temperature of digestion tanks reducing to room temperature, tanks were slowly unscrewed to drive away the acid, then all the solution was transferred into the volumetric flask (50 mL). All the volumetric flasks were titrated to 50 mL with deionized water, and then homogenized for being measured.

### Determination by Flame Atomic Absorption Spectrometry (FAAS)

A fast sequential FAAS (Jena Analytical Instruments Co., Ltd, Germany) was employed to carry out all measurements. Pb^2+^ hollow cathode lamps were used as radiation sources. An air/acetylene flame was used for Pb^2+^ element determinations. The FAAS operating conditions were 0.4 nm Slit width, 2.0 mA, Applied current, 1.0 nm Spectral resolution, 1700 L min^−1^ Acetylene flow rate.

Pb^2+^ standard solution samples of 0 mg/L, 0.05 mg/L, 0.1 mg/L, 0.2 mg/L, 0.4 mg/L, 0.8 mg/L (the standard series) was prepared to draw the Pb^2+^ standard curve, after determination by FAAS.

KI, AA and MIBK were used as extractants. 1.5 mL KI, 1 mL AA and 5 mL MIBK in sequence was added to the sample digestion solution, the reagent blank and the standard series. Each time an extractant was added, the sample was oscillated for 2 min.

After the extraction of 10 min, the sample was 200 times diluted, and then the organic phase was introduced into the FAAS. Determination of absorbance at 283.3 nm wavelengths, a standard curve or calculate a linear regression equation was drew with the standard series of absorbance. The absorbance of the sample was brought into the equation to obtain the residual Pb^2+^ concentration.

### The adsorption capacity of bacteria

The adsorption capacity of the strains towards Pb^2+^ was expressed as the removed rate and bacterial adsorption capacity (Baig et al. 2010).

$${\text{The removal rate }}\left( \% \right)\, = \,\frac{{C_{0} - C_{e} }}{{C_{0} }} \times 100$$

C_o_ is the initial Pb^2+^ concentration, C_e_ is the residual Pb^2+^ concentration after removal.

$$Q_{e} = \frac{{(C_{0} - C_{e} ) \times V}}{m}$$

Q_e_: Bacterial adsorption capacity, C_o_ represents the initial Pb^2+^ concentration, C_e_ represents the equilibrium solution of Pb^2+^ concentration. V: the volume of Pb^2+^ solution, m: weight of wet cell, m/V = 3 g/L.

### Statistical analysis

All the experiments were carried out in triplicate, and the results are provided as the mean ± SD (standard deviation) values. One-way analysis of variance (ANOVA) was performed, and the significance of each mean property value was determined (p < 0.05) with Duncan’s multiple range test with SPSS software (SPSS Inc., IL, USA).

## Result

### Colony morphology

**Fig. 1:**
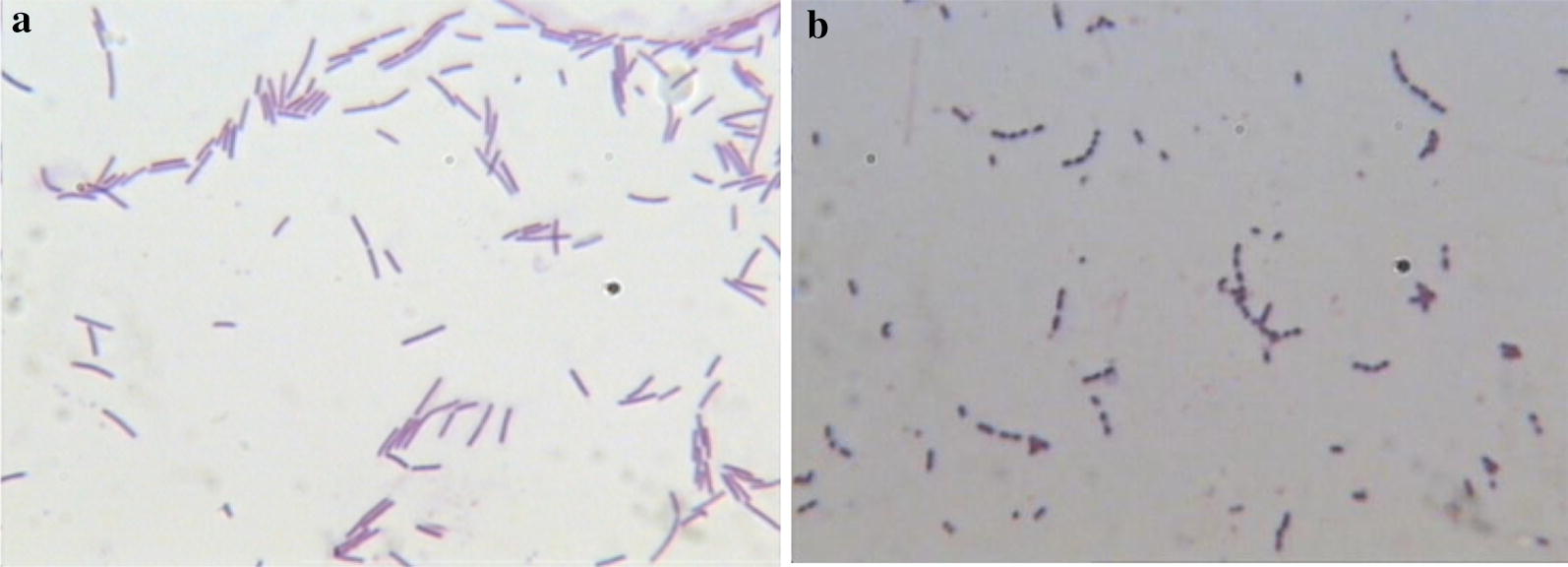
Gram staining and microscopy of colonies. **a** Represents TMP and **b** represents TSP

As could be seen from Fig. 1, most of the bacteria were stalked, single or chain. However, the cell size of bacteria shrank obviously and the morphology atrophied with the rod structure absent, which could be seen in Fig. 1b. The Pb^2+^ on TSP inhibited the accumulation of bacterial components (Goswami et al. 2017) or changed  the synthesis of cell walls (Xu et al. 2016), leading to the change in the morphology of the bacteria (Li et al. 2017).

Morphological characteristics were compared between TMP and TSP (Table 1). The colony on TMP was generally larger than TSP’s. The colony morphologies of TMP and TSP both were white, smooth surface, tidy edge and round colony with different size. The colony on TMP grew well, showing a normal growth state of bacteria, while the colonies on TSP became very small and transparent, which showed that Pb^2+^ has a great effect on the growth of bacteria. The colony on TMP was generally larger than TSP’s. Combined with Fig. 1, the mutation of bacteria was further determined.

**Table 1:** Colony morphology comparison of TMP and TSP

	TMP	TSP
Image		
Size	Medium-sized, 1–5 mm diameter	Small, compact arrangement
Form	Round, regular edges	Round, regular edges
Protruding	Slightly bulging	Raised
Surface state	Smooth	Smooth
Surface gloss	Glossy	Little glossy
Texture	Wet and sticky, easy to pick	Sticky, difficult to pick
Color and transparency	Thick and white	White and transparent

According to Bergey’ s manual of systematic bacteriology (Peladan and Monteil 1984), some suspected LAB strains were inoculated on TSP for screening Pb^2+^ resistant strains. In the end, 32 strains were selected from TSP and marked from LAB-01 to LAB-32.

### Acclimatization of bacteria

All the 32 strains could grow on the Pb^2+^ medium, but with the increase of Pb^2+^ concentration, the number of colonies with morphological variation increased (Table 2). At the same time, the culture time of the strains in MRS ager medium was about 32 h, but when Pb^2+^ was added into the medium, the strains had a prolonged adaptation period and a significant decrease number of colonies. Pb^2+^ could decrease the growth rate of bacteria by regulating or inhibiting some enzymatic reactions or by binding with DNA, membranes and cell walls (Kurniawan et al. 2018). In present experiment, the stable growth time of the colony was 48 h.

**Table 2:** Colony morphology of Pb^2+^ medium

500 mg/L	1000 mg/L	1500 mg/L	2000 mg/L
			
Well growth	Slow growth	Slow growth	Slow growth and some variation

Finally, 7 strains of Pb^2+^-resistance strains (LAB-05, LAB-08, LAB-13 LAB-32 LAB-10, LAB-23, LAB-27) were preserved for identification.

### Identification of strains

**Fig. 2:**
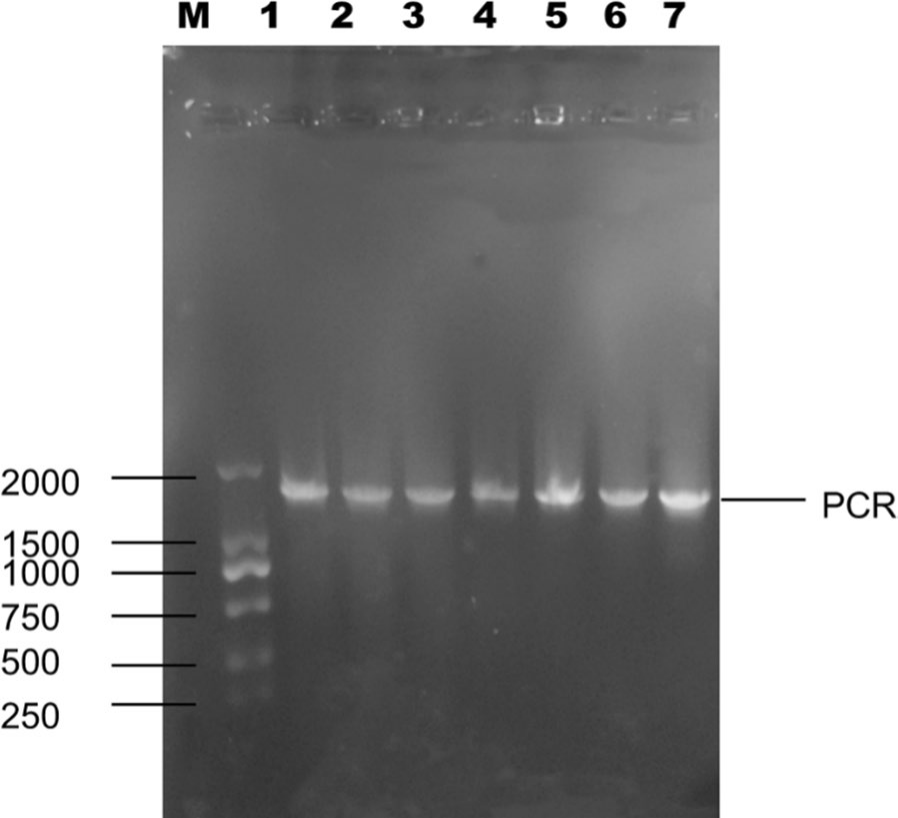
Electrophoresis of PCR-amplified 16 S rDNA gene fragments from 7 strains (left to right: Marker, 05, 08, 13, 23, 27, 10, 32)

According to the contrast of PCR products detected by 1% agarose gel electrophoresis between Marker and 7 strains (Fig. 2), specific bands of 7 strains were found at 1500 bp**–**2000 bp, which conformed to expectation, indicating that the PCR amplification was successful.

**Fig. 3:**
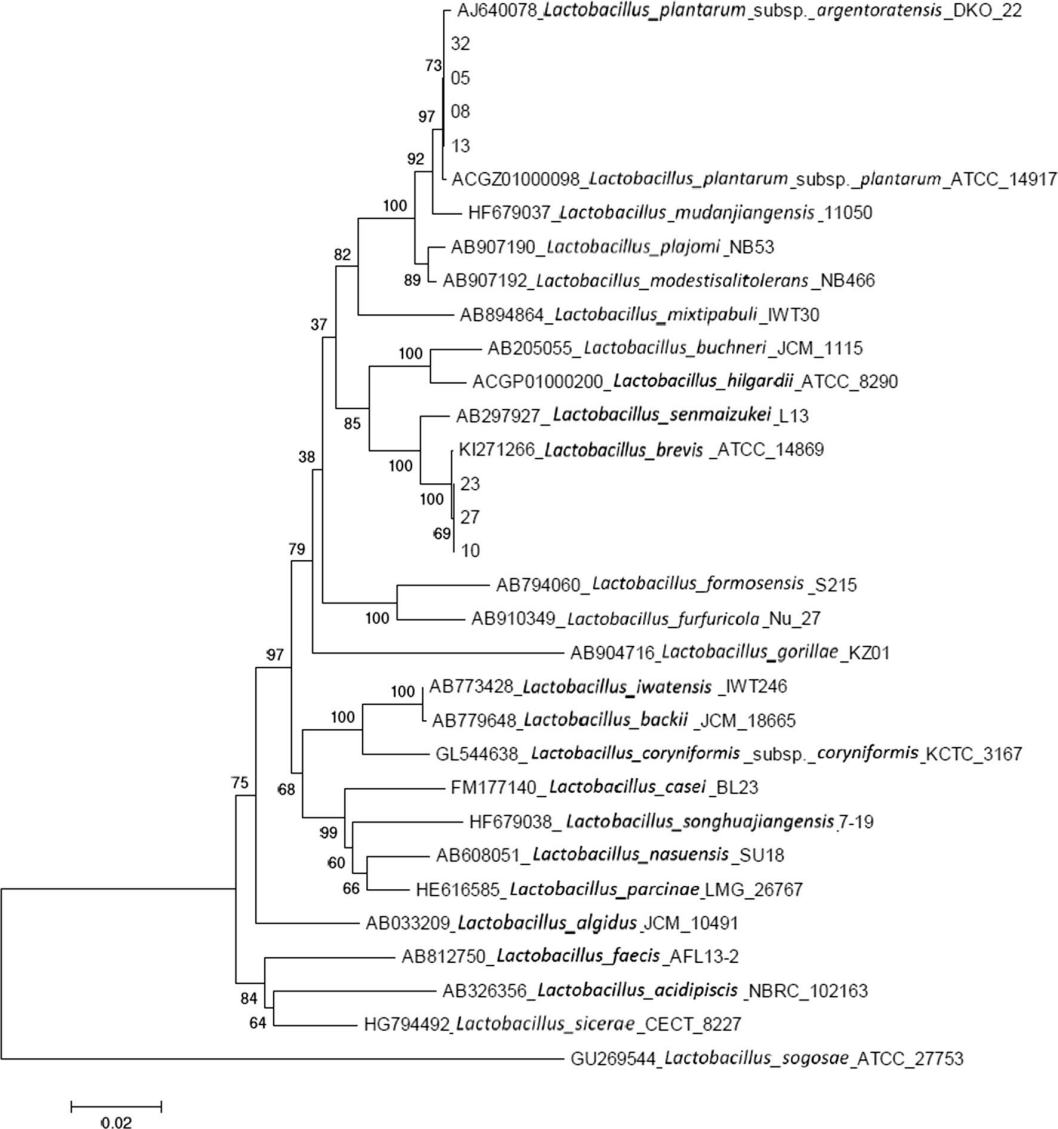
Phylogenetic tree based on 16 S-rDNA sequences of 7 strains

After sequencing, the 16S-rDNA gene sequence of each strain was carried out BLAST analysis in GenBank. To show the relationship between strains, the phylogenetic tree (Fig. 3) was constructed by using MEGA 5.1 software based on 16 s rDNA gene sequence extracted from those LAB and GenBank database (Alexander et al. 2014).

Computational integration of genomic traits into 16S rDNA microbiota sequencing was studied. The homologies of the 7 strains and the reference bacteria were more than 97%. When sequence homologies of 16S rDNA were over 97%, it could be considered to belong to the same genus (Auch et al. 2010). Thereafter, 05, 08, 13 and 32 belong to a same genus, 10, 23 and 27 belong to a same genus. As Fig. 3 shown, 05, 08, 13 and 32 were closely related to *L. plantarum*, and 10, 23 and 27 were closely related to *Lactobacillus (L. brevis).* The similarity rate of two groups to their similar bacteria was over 97%. The genus of the 7 strains of LAB were shown in Table 3.

**Table 3:** The genus of 7 strains

LAB	Genus
05	*Lactobacillus plantarum*
08	*Lactobacillus plantarum*
10	*Lactobacillus brevis*
13	*Lactobacillus plantarum*
23	*Lactobacillus brevis*
27	*Lactobacillus brevis*
32	*Lactobacillus plantarum*

### Optimization of digestion conditions

As could be seen from Table 4 that the second method was digested fully and sufficiently. HNO_3_ had strong oxidation property, and was easily vaporized acid after digestion. Therefore, 0.5 mL of sample, 6 mL of HNO_3_ and 2 mL of H_2_O_2 _were taken.

**Table 4:** Digestion degree of different proportion of solvent

No.	Degree of digestion
One	+ + + + +
Two	+ + + +
Three	+ + +

### Adsorption capacity of LAB

**Fig. 4:**
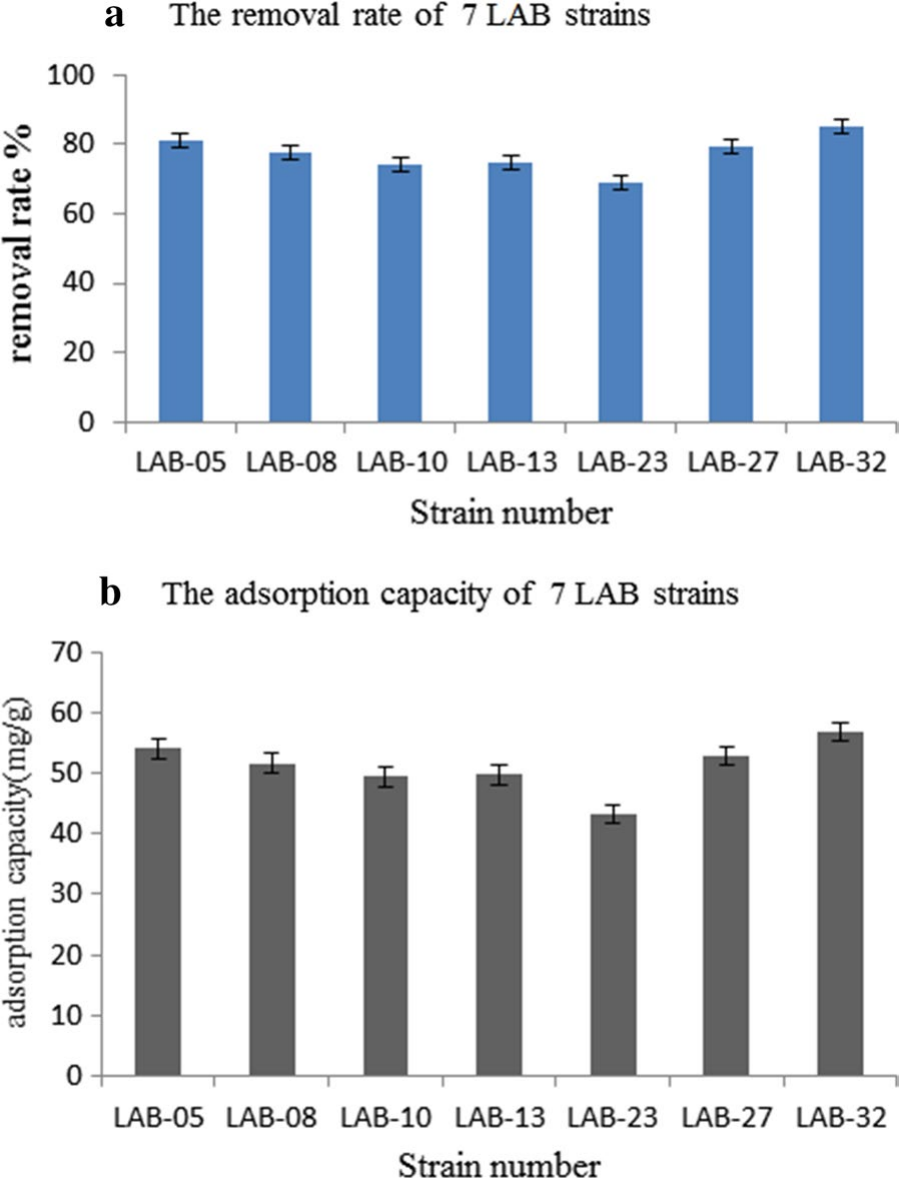
The removal rate (**a**) and adsorption capacity (**b**) of 7 LAB strains. The blue bar chart represents the removal rate and the gray bar chart represents the adsorption amount

As could be seen from Fig. 4, the removal rate of the 7 LAB strains, of which LAB-32 had the highest removal rate and its average adsorption capacity reached 54.03 mg/g, LAB-23 had the lowest removal rate and its average adsorption capacity reached 43.26 mg/g. Besides, LAB-08, LAB-10 and LAB-13 had similar adsorption capacity.

At present, there were few reports on the adsorption of LAB towards Pb^2+^, especially for *L. plantarum* and *L. brevis*, which were almost not reported. The adsorbents of Pb^2+^ were divided into non-biological adsorbent and biological adsorbent, different kinds of adsorbents had different removal rates (Table 5).

**Table 5:** The removal rate of different material towards Pb^2+^

Type of adsorbent	Adsorbent	Maximum adsorption (mg/g)	References
Non-biological adsorbent	Coconut-shell carbon (dry) freshbrine algae	26.50	Sekar et al. (2004)
	Original diatomite (DO)	3.1101	Sögüt and Caliskana (2017)
	Manganese oxide modified Diatomite (DMn)	12.6322	
	Sawdust	4.59	Li et al. (2007)
	Modified peanut husk	4.66	
	Acidified multi-walled CNTs	49.71	Wang et al. (2007)
	Original bentonite	19.19	Kul and Koyuncu (2010)
	Cicer arientinum biomass	27.79	Nadeem et al. (2009)
	Coontail or hornwort	44.8	Keskinkan et al. (2004)
	Pine wood char	4.13	Mohan et al. (2007)
	ACF-400	30.11	
Biological adsorbent	Oedogonium hatei	40.9–44.2	Gupta et al. (2010)
	Derived vermicompost (CV)	38.11	Zhu et al. (2017)
	Cow manure (CM)	43.01	
	*L. rhamnosus*- GG	46.8	Halttunen et al. (2007)
	*B. longum* 2C	45.4	
	*L. rhamnosus* LC-705	29.1	Ibrahim et al. (2006)
	LAB-05 (*Lactobacillus plantarum*)	55.31	This work
	LAB-08 (*Lactobacillus plantarum*)	51.83	This work
	LAB-10 (*Lactobacillus brevis*)	50.31	This work
	LAB-13 (*Lactobacillus plantarum*)	50.94	This work
	LAB-23 (*Lactobacillus brevis*)	44.17	This work
	LAB-27 (*Lactobacillus brevis*)	53.71	This work
	LAB-32 (*Lactobacillus plantarum*)	57.31	This work

As can be seen from the comparison in Table 5, in general, the adsorption capacity of 7 strains in different adsorbents was at a high level. In the non-biological adsorbent, the maximum adsorption capacity was 49.71 mg/g, while in the biological adsorbent, the maximum adsorption capacity reached 55.72 mg/g.

## Discussion

**Fig. 5:**
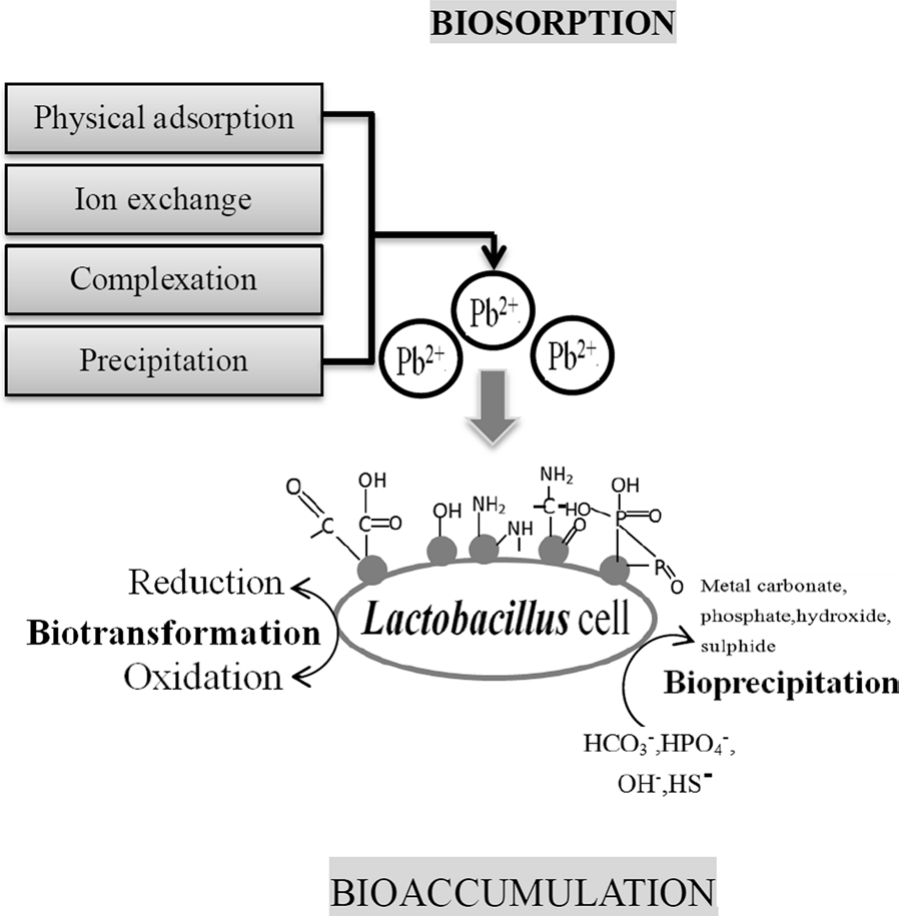
The mechanism of biosorption and bioaccumulation of a microbial cell

Due to the high adsorption capacities of 7 strains to Pb^2+^, we referred to existing research results to speculate on the process of adsorption of Pb^2+^ by LAB in this experiment (Fig. 5).

Metal ions enter the living LAB usually through extracellular biosorption and intracellular bioaccumulation (Huang et al. 2018). Biological adsorption mainly refers to the adsorption of metal ions onto biological matrix by one or more combination of includes physical adsorption, precipitation, complexation, coordination, chelation, ion exchange, electrostatic interaction and microprecipitation (Vijayaraghavan and Yun 2008; Diep et al. 2018). Bioaccumulation includes cytoplasmic sequestration, enzymatic detoxification and trafficking to efflux systems (Huang et al. 2018). The former was a fast process, which took only dozens of minutes without consuming energy. But bioaccumulation was a metabolic process that requires energy. Microorganisms were really sensitive to heavy metal stress. Heavy metals show the highest toxicity to cell, exhibiting an increase in the production of apoptosis and cell death. The damage of the DNA of bacteria were negatively correlated with adsorption ability of bacteria (Zhou et al. 2008; Giller et al. 1998). Therefore, the abnormal situation in this experiment was closely related to the toxicity and stress of heavy metals. The deeper reason may be the damage to bacteria DNA and other substances caused by heavy metals.

Biological materials had good biosorption capacity for metal ions (Vijayaraghavan and Yun 2008). Compared with abiotic adsorbent, the effect of biological adsorbents was more stable, more widely used and cheaper (Vinod et al. 2015). The bacterial cell wall was the first component in contact with metal ions, which were deposited first on the cell surface or in the cell wall structure. The chemical functional groups of the cell wall played a vital role in biosorption (Vijayaraghavan and Yun 2008), such as carboxylate, hydroxyl, sulfate, phosphate and amino, etc (François, et al. 2012). Sintuprapa et al. (2000) suggested that the functional groups of cell wall containing negative charges in living cells of *Penicillium sp.*, such as phosphate groups, hydroxyl groups and carboxyl groups, reacted with Zn^2+^ to adsorb Zn^2+^. Then Zn^2+^ bound to polyphosphate granules and accumulated and precipitated in cells. Feng et al. (2012) isolated Pb^2+^-resistant *train Lb. plantarum 70810 EPS* from traditional Chinese pickled cabbage, they observed a large number of Pb^2+^ particles adsorbed on the surface of cell by scanning electron microscopy, which showed that the functional groups joining in adsorption involve the hydroxyl group, carboxyl group, sulfate group, amino group and amide group and so on. In this study, LAB mainly relied on cell surface adsorption to blind free Pb^2+^ through positive and negative charges to the surface of cell wall. The cell wall of LAB was mainly composed of mannan, glucan, chitin and protein. The hydroxyl group, carboxyl group, sulfate group, amino group and amide group in these components can be complex with Pb^2+^, then retained by mineral nucleation (Murthy et al. 2012). In addition, cell wall peptidoglycans and/or surface extracellular polymers with cell wall dissociation could also form effective biosorption matrices to adsorb metal ions (Vijayaraghavan and Yun 2008). Gram-positive microorganisms had a large adsorption capacity because they have a thick peptidoglycan layer and contain a large number of adsorption sites (Timková et al. 2018). François et al. (2012) also confirmed that the biosorption of mercury by *Bacillus sp. CM111* depends on the surface extracellular polymeric substances. Similarly, the extracellular polymeric substances of LAB could be used as metal ion binders to adsorb Pb^2+^. Metal ions and bacterial cell surface could be combined by electrostatic interaction, van der Waals force, covalent bond, redox interaction and precipitation (Murthy et al. 2012). 

Due to all the samples in this study were exerted shake cultivation for 1 h, which was a relatively short time. A large number of Pb^2+^ could be adsorbed in a short time, and extracellular biosorption played the most important role, followed by bioaccumulation.

Bioaccumulation is a metabolic process in which metal ions are absorbed into the intracellular space by the import complexes which create a translocation pathway through the lipid bilayer and then sequestered by the protein and peptide ligands (Diep et al. 2018). Bioaccumulation is a process after biosorption (Christoforidis et al. 2015). When most of places on the cell wall that can be used for metal ion binding are occupied, living microbial cells initiated intracellular bioaccumulation (Mrvčić et al. 2012). After Pb^2+^ was transported inside LAB, there were two possible ways to explain bioaccumulation. For one thing, Pb^2+^ was transported to the cytosol and isolated in certain areas to prevent damage to important organelles and functional molecules. For another, Pb^2+^ bound to the Pb^2+^-binding protein, which reduced the toxicity of Pb^2+^ and kept the cells growing normally. It was obvious that the reaction of Pb^2+^ and binding proteins was irreversible. Bioaccumulation of Pb^2+^ in cells depended on the growth of LAB (Podder and Majumder 2018).

In addition, pH, temperature, pretreatment, initial metal ion concentration, contact time, interfering ions, biomass concentration and other factors also had different impact on the adsorption capacity of LAB (Lin et al. 2017).

Adsorption capacity of* L. brevis* and *L. plantarum* was studied under the optimum growth conditions of LAB, but not under their own optimum growth conditions. If under those conditions, the adsorption capacity of them should be improved. So, next, we would continue to study the optimal growth conditions of these two strains of LAB and used them separately or jointly to solve the problem of Pb^2+^ contamination in food.

In general, this study was to screen the Pb^2+^ resistant LAB from Ya’an pickle brine. 7 strains with strong tolerance to Pb^2+^ were isolated for identification. The results showed that 4 strains of *L. plantarum* and 3 strains of *L.s brevis* were obtained in the 7 strains of LAB. Through the blinding experiment, their adsorption capacities towards Pb^2+^ were at 43.26–56.83 mg/g, which were at a higher level. Through the explanation of the adsorption mechanism of LAB, the adsorption of LAB towards Pb^2+^ mainly involved biosorption and bioaccumulation. The 7 strains of LAB mainly relied on biosorption to remove Pb^2+^, at the same time, the complexation on the bacterial surface played a decisive impact. The 7 strains of LAB could be great potential as a safe adsorbent to solve heavy metal pollution in the food and the accumulation of heavy metals in human body.

## Additional file

**Additional file 1: Table S1.** Microwave digestion program. **Table S2.** The proportion of solvent.
