# Supplementary material for: Screening and identification of Lactic acid bacteria from Ya’an pickle water to effectively remove Pb2+
Source: AMB Express. 2019 Jan 19;9:10. doi: 10.1186/s13568-018-0724-y (PMC6339634; doi:10.1186/s13568-018-0724-y)
Supplement: Supplementary file 1 — Additional file 1: Table S1. Microwave digestion program. Table S2. The proportion of solvent. [file 13568_2018_724_MOESM1_ESM.doc]

Table S1. Microwave digestion program

| Program order | Ramp time  （min） | Temperature  （℃） | Pressure  (M Pa) | Hold time  （min） |
| --- | --- | --- | --- | --- |
| 1 | 5 | 140 | 6.0 | 5 |
| 2 | 10 | 170 | 6.0 | 10 |
| 3 | 5 | 100 | 6.0 | 5 |

Table S2. The proportion of solvent

| No. | Sample  (mL) | HNO_3_  (mL) | H_2_O_2_  (mL) |
| --- | --- | --- | --- |
| One | 0.5 | 6 | 2 |
|  | 0.5 | 5 | 3 |
| Two | 0.5 | 4 | 4 |
|  | 1.0 | 6 | 2 |
| Three | 1.0 | 5 | 3 |
|  | 1.0 | 4 | 4 |
